# High Prevalence of Virulence Genes in Specific Genotypes of Atypical Enteropathogenic *Escherichia coli*

**DOI:** 10.3389/fcimb.2017.00109

**Published:** 2017-04-04

**Authors:** Yanmei Xu, Xiangning Bai, Yujuan Jin, Bin Hu, Hong Wang, Hui Sun, Ruyue Fan, Shanshan Fu, Yanwen Xiong

**Affiliations:** ^1^State Key Laboratory of Infectious Disease Prevention and Control, Collaborative Innovation Center for Diagnosis and Treatment of Infectious Diseases, National Institute for Communicable Disease Control and Prevention, Chinese Center for Disease Control and PreventionBeijing, China; ^2^Longgang Center for Disease Control and PreventionShenzhen, China; ^3^Shandong Center for Disease Control and PreventionJinan, China; ^4^Zigong Center for Disease Control and PreventionZigong, China

**Keywords:** *E*. *coli*, EPEC, serotyping, MLST, virulence gene

## Abstract

Atypical enteropathogenic *Escherichia coli* (aEPEC) strains are emerging enteropathogens that have been detected worldwide. A collection of 228 aEPEC strains (121 from diarrheal patients, 27 from healthy carriers, 47 from animals and 33 from raw meats) were investigated for serotypes, virulence gene profiles and phylogenetic relationships. Sixty-six O serogroups were identified. Serogroup O51 was the most prevalent, followed by O119, O26 and O76. For the 20 virulence genes detected, statistically significant differences were observed in the overall prevalence of *efa1* (*lifA*), *nleB, nleE, set*/*ent, paa*, and *ehxA* genes among strains from diarrheal patients, healthy carriers, animals and raw meats, respectively. Strains from diarrheal patients had significantly higher levels of *efa1* (*lifA*) (29.8 vs. 0%, *P* = 0.0002), *nleB* (41.3 vs. 7.4%, *P* = 0.0004), *nleE* (43.8 vs. 7.4%, *P* = 0.0002) and *set/ent* (41.3 vs. 7.4%, *P* = 0.0004) genes than strains obtained from healthy carriers. The *paa* gene was identified more often in isolates from raw meats (63.6 vs. 14.8%, *P* < 0.0001), animals (42.6 vs. 14.8%, *P* < 0.0122), and diarrheal patients (36.4 vs. 14.8%, *P* < 0.0225) than in strains obtained from healthy carriers. The *ehxA* gene was detected more frequently in strains from raw meats than in strains from diarrheal patients (27.3 vs. 2.5%, *P* = 0.0000) and healthy carriers (27.3 vs. 7.4%, *P* = 0.0474). The phylogenetic marker, *yjaA*, was more frequently observed in strains among healthy carriers than in diarrheal patient strains. Among the 228 aEPEC strains, 79 sequence types (STs) were identified. The prominent STs, which comprised strains carrying the four OI-122 genes and *lpfA*, were ST40, ST328, and ST29. Overall, the results indicate that aEPEC strains isolated in China are highly heterogeneous. aEPEC strains that are potentially more pathogenic appear to be related to specific STs or clonal complexes and serotypes. The high prevalence of diarrhea-associated genes in animal or raw meat strains suggests a zoonotic transmission pathway for potentially human pathogenic aEPEC.

## Introduction

Globally, one in ten childhood deaths are due to diarrheal disease among children under 5 years old, leading to about 800,000 fatalities worldwide annually, most of which occur in areas of sub-Saharan Africa and south Asia (Kotloff et al., 2013). Enteropathogenic *Escherichia coli* (EPEC), the first pathogenic type of *E. coli* to be associated with human disease, is one of the most prevalent pathogen infecting children worldwide (Ochoa and Contreras, 2011). EPEC strains are able to form attaching and effacing (A/E) lesions through intimate adherence and effacement of the intestinal microvilli of the host. A/E lesion production is dependent on the pathogenicity island LEE (locus of enterocyte effacement), which is integral to EPEC pathogenicity. Based on the presence or absence of the EPEC adherence factor plasmid (pEAF), EPEC may be further classified as typical EPEC (tEPEC) or atypical EPEC (aEPEC), respectively (Croxen et al., 2013). In the last 10 years, epidemiological studies of tEPEC and aEPEC have shown a much higher prevalence of aEPEC in both developed and developing countries, and aEPEC has emerged as an important pathogen (Hu and Torres, 2015). We, and other investigators, have clearly shown its high prevalence in China.

The majority of tEPEC strains produce virulence traits on LEE and pEAF, while aEPEC strains mostly have both additional and heterogeneous virulence properties (Hu and Torres, 2015). Bando et al. found that aEPEC strains showed genetic similarity with other diarrheagenic *E. coli* (DEC) pathotypes and contained virulence factors derived from other DEC pathotypes. So they inferred that aEPEC strains might have a particular genetic background that allows the acquisition and expression of virulent genes derived from other pathotypes (Bando et al., 2009). As a consequence, aEPEC strains are phylogenetically heterogeneous and carry virulence factors of other pathogenic *E. coli* more often than tEPEC strains (Hernandes et al., 2009). However, the virulence determinants linked with aEPEC infection have yet to be determined.

Besides the LEE region, another pathogenicity island (PAI), OI-122 has been described in aEPEC. This PAI harbors genes *efa1 (lifA)* that encodes lymphocyte inhibitory factor (LifA) (Abu-Median et al., 2006), *set/ent* that encodes a homologous *Shigella flexneri* enterotoxin (Nataro et al., 1995), and *nleB* and *nleE* genes that encode proteins, which inhibit pro-inflammatory signaling (Newton et al., 2010). The simultaneous presence of all the OI-122 genes i.e., *efa1* (*lifA*), *set/ent, nleB*, and *nleE*, is statistically associated with diarrhea compared to healthy controls (Afset et al., 2006; Mercado et al., 2016).

In addition to the LEE-encoded factors, other virulence determinants may contribute to the pathogenesis of aEPEC. These include *astA*, which encodes the enteroaggregative heat-stable toxin 1 (EAST1), and has been shown to be significantly associated with diarrhea in a case-control study in Brazil (Dulguer et al., 2003) and a waterborne outbreak of diarrhea in Japan (Yatsuyanagi et al., 2003). Two toxins can cause apoptosis: cytolethal distending toxin (CDT) which causes characteristic and irreversible cell cycle arrest (Jinadasa et al., 2011), and subtilase cytotoxin (SubAB) which triggers endoplasmic reticulum stress signaling pathways leading to apoptosis (Paton and Paton, 2010). Autotransporters are often associated with virulence functions such as adherence, aggregation, invasion, biofilm formation, and toxicity (Abreu et al., 2013). Pathogenesis is mediated by the plasmid encoded toxin (Pet) (Ruiz et al., 2014), the Pic serine protease (Abreu et al., 2016), and an extracellular serine protease (EspP) (Brunder et al., 1999). Antiaggregation protein (dispersin), encoded by the *aap* gene, acts by dispersing the pathogen through the mucus layer produced by the intestinal epithelial cells, and is translocated by the antiaggregation transporter protein encoded by *aat* (Sheikh et al., 2002). Other putative virulence factors such as an enterohemolysin (EhxA) and a catalase/peroxidase (KatP), encoded by the genes located in a megaplasmid of Shiga toxin-producing *E. coli* (STEC) have also been reported (Kobayashi et al., 2013). The phylogenetic marker gene *yjaA*, was reported to be negatively associated with diarrhea (Wang et al., 2013).

The first step in gastrointestinal infection is the initial attachment to the surface of the host intestinal epithelium (Kalita et al., 2014). Several adherence-related factors have been described as being present in EPEC or STEC isolates, such as, the enterohemorrhagic *E. coli* (EHEC) autotransporter C, encoded by *ehaC* (Abreu et al., 2013); the Iha virulence factor, encoded by *iha* (Tarr et al., 2000; Bardiau et al., 2009); ToxB, encoded by *toxB* on the pO157 plasmid (Tatsuno et al., 2001); an autoagglutinating adhesin, Saa (Paton et al., 2001); porcine attaching and effacing-associated adhesin (Paa), encoded by *paa* (Batisson et al., 2003; Maluta et al., 2014); long polar fimbria (LPF) (Bardiau et al., 2010).

Since tEPEC strains are rarely isolated from animals, humans are generally considered to be the main reservoir of tEPEC. Whereas aEPEC are present in both healthy and diseased animals and humans (Hernandes et al., 2009; Hu and Torres, 2015). In the current investigation, we characterized 228 aEPEC strains recovered from multiple sources (diarrheal patients, healthy carriers, animals and raw meats), and attempted to investigate their molecular characteristics, phylogenetic relationship, and understand the potential factors involved in human infection.

## Materials and methods

### Bacterial strains

In a previous study, a total of 3401 specimens from different sources were collected from 2006 to 2015 in seven geographical regions in China. In which, stool samples of diarrheal patients were collected during clinical treatment at sentinel hospitals, fecal samples of healthy humans (without diarrhea in recent 2 weeks) were collected during routine physical examination, and stool samples of animals (cattle, pig, live chicken, bird, marmota, ochtona) and raw meat samples (chicken meat, beef, pork, mutton) were collected in routine surveys (Xu et al., 2016). Briefly, 1.5 ml of each enrichment [cultured overnight in modified Tryptone Soya Broth (mTSB) (Oxoid, UK)] was centrifuged. Then, 150 μl of the lysis buffer (100 mM NaCl, 10 mM Tris–HCl [pH 8.3], 1 mM EDTA [pH 9.0], 1% Triton X-100) was added, resuspended, boiled and centrifuged. The released DNA were subjected to PCR assay for eae gene (Hu and Torres, 2015). The *eae*-positive enrichment culture were inoculated onto CHROMagar™ ECC plate (CHROMagar, France) and further processed for isolation of aEPEC as previously described (Xu et al., 2016). The *eae*-positive colonies were subcultured on Luria-Bertani (Oxoid, UK) plates and incubated for further confirmation by standard biochemical tests. Isolates with *eae*^+^, stx1/stx2^−^, and bfpA^−^ were confirmed as aEPEC (Hu and Torres, 2015). Only one isolate from each sample was kept for further analysis.

Totally, 228 aEPEC isolates were identified during the epidemiological studies. 121, 27, and 47 strains were isolated from the fecal samples of diarrheal patients, healthy carriers and animals, respectively. The remaining 33 isolates were recovered from raw meats. Bacteria were stored at −80°C before being subcultured aerobically at 37°C on Luria-Bertani (LB) agar at 37°C.

### Extraction of DNA from aEPEC isolates

To prepare template DNA for PCR, the distilled water-boiling method was employed (Ooka et al., 2009). A single colony from the aEPEC isolates was suspended in 100 μl of deionized water and boiled for 10 min. After being centrifuged at 10,000 × g for 5 min, the supernatant containing DNA was collected, and 1 μl of the supernatant was used as template DNA.

### Serotyping of aEPEC isolates

The O serogroups were screened by the PCR-based system arranged by Iguchi et al. (2015). The PCR results were confirmed using complete *E. coli* O antisera (Statens Serum Institute, Denmark). The isolates were defined as O-untypable if they did not react with any O antisera. H typing was performed by PCR amplification and sequencing of the *fliC* gene using primers fliC-F (5′-ATGGCACAAGTCATTAATACCCAAC-3′) and fliC-R (5′-CTAACCCTGCAGCAGAGACA-3′) (Fields et al., 1997). These were then compared to the known variants of *fliC* gene on the SerotypeFinder database (https://cge.cbs.dtu.dk/services/SerotypeFinder/) (Joensen et al., 2015). The isolate was considered as H-untypable if *fliC* was negative by PCR.

### Detection of virulence genes by PCR

The 228 aEPEC strains were investigated by PCR for the presence of putative virulence genes or adhesion genes (*ehxA, subAB, nleB, nleE, set/ent, espP, katP, astA, pet, aat, ehaC, pic, aap* and *cdt, iha, efa1, lfpA, saa, toxB*, and *paa*) and a phylogenetic marker gene *yja*. Three variants (*lfpA*_*O113*_, *lfpA*_*O157-154*_, *lfpAO*_*157-141*_) of the LPF encoding gene (*lpfA*) and two variants (*cdt1, cdt2*) of the *cdt* gene were included. PCR amplifications were performed in a thermal cycler (SensoQuest Labcycler, Germany). PCR cycling was performed according to the following conditions: initial denaturation at 95°C for 5 min, 30 cycles, each of denaturation at 95°C for 1 min, then annealing at the corresponding annealing temperature of 1 min and nucleotide extension at 72°C for 1 min. All primers, sizes of amplified fragments and PCR conditions used for this screening and corresponding references are shown in Table 1. Amplified products were analyzed by 1.5% agarose gel electrophoresis.

**Table 1:** **PCR primers used for the detection of putative virulence or adherence genes**.

**Genes**	**Primer**	**Oligonucleotide sequence (5′–3′)**	**Amplicon size (bp)**	**Annealing Temp (°C)**	**References**
*efa1 (lifA)*	*efa1*-F	AAGGTGTTACAGAGATTA	266	51	Nicholls et al., 2000
	*efa1*-R	TGAGGCGGCAGGATAGTT			
*set*	*set*-F	TTCCTGGGTTGCTTTTAGCTCT	171	60	Wang et al., 2013
	*set*-R	CATGTCCATTTTGAAGGGCCTG			
*nleB*	*nleB*-F	GGTGTGCTGGTAGATGGA	175	53	Afset et al., 2006
	*nleB*-R	CAGGGTATGATTCTTGTTTATG			
*nleE*	*nleE*-F	CTAATACTCAGGGCGTGTCC	192	53	Afset et al., 2006
	*nleE*-R	ACCGTCTGGCTTTCTCGTTA			
*lpfA*_*O*113_	*lpfA*_*O*113_ –F	ATGAAGCGTAATATTATAG	573	50	Afset et al., 2006
	*lpfA*_*O*113_ –R	TTATTTCTTATATTCGAC			
*lpfA*_*O*157/*OI*−154_	OI-154-F	GCAGGTCACCTACAGGCGGC	525	55	Toma et al., 2004
	OI-154-R	CTGCGAGTCGGCGTTAGCTG			
*lpfA_*O*157/*OI*−141_*	OI-141-F	CTGCGCATTGCCGTAAC	412	54	Szalo et al., 2002
	OI-141-R	ATTTACAGGCGAGATCGTG			
*paa*	*paa*–F	ATGAGGAACATAATGGCAGG	360	55	Afset et al., 2006
	*paa*–R	TCTGGTCAGGTCGTCAATAC			
*iha*	*iha*–F	CAGTTCAGTTTCGCATTCACC	1,305	56	Schmidt et al., 2001
	*iha*-R	GTATGGCTCTGATGCGATG			
*saa*	*saa*-F	CGTGATGAACAGGCTATTGC	119	52	Paton and Paton, 2002
	*saa*-R	ATGGACATGCCTGTGGCAAC			
*pet*	*pet*-F	GGCACAGAATAAAGGGGTGTTT	302	62	Patzi-Vargas et al., 2015
	*pet*-R	CCTCTTGTTTCCACGACATAC			
*aat*	*aat*-F	CTGGCGAAAGACTGTATCAT	629	55	Patzi-Vargas et al., 2015
	*aat*-R	CAATGTATAGAAATCCGCTGTT			
*astA*	*astA*-F	GCCATCAACACAGTATATCC	111	62	Patzi-Vargas et al., 2015
	*astA*-R	GAGTGACGGCTTTGTAGTC			
*aap*	*aap*-F	CTTGGGTATCAGCCTGAATG	310	55	Patzi-Vargas et al., 2015
	*aap*-R	AACCCATTCGGTTAGAGCAC			
*cdt1*	*CDT-Is*	CAATAGTCGCCCACAGGA	411	55	Patzi-Vargas et al., 2015
	*CDT-Ias*	ATAATCAAGAACACCACCAC			
*cdt2*	*CDT-IIs*	GAAAGTAAATGGAATATAAATGTCCG	556	55	Patzi-Vargas et al., 2015
	*CDT-IIas*	TTTGTGTTGCCGCCGCTGGTGAAA			
*subAB*	*subAB*-F	TATGGCTTCCCTCATTGCC	556	55	Patzi-Vargas et al., 2015
	*subAB*-R	TATAGCTGTTGCTTCTGACG			
*ehxA*	*ehxA*-F	GGTGCAGCAGAAAAAGTTGTAG	1,551	57	Bai et al., 2013
	*ehxA*-R	TCTCGCCTGATAGTGTTTGGTA			
*katP*	*katP*-F	CTTCCTGTTCTGATTCTTCTGG	2,125	56	Brunder et al., 1996
	*katP*-R	AACTTATTTCTCGCATCATCC			
*espP*	*espP*	AAACAGCAGGCACTTGAACG	1,830	56	Bai et al., 2013
	*espP*	GGAGTCGTCAGTCAGTAGAT			
*toxB*	*toxB*-F	ATACCTACCTGCTCTGGATTGA	602	55	Tarr et al., 2002
	*toxB*-R	TTCTTACCTGATCTGATGCAGC			
*pic*	*pic*-F	GGGTATTGTCCGTTCCGAT	1,176	60	Abreu et al., 2013
	*pic*-R	ACAACGATACCGTCTCCCG			
*ehaC*	*ehaC*-F	TAATGACGGCAAAGGTGGT	599	59	Abreu et al., 2013
	*ehaC*-R	CATTCATCAGGGAGTTGCT			
*yjaA*	*yjaA*-F	TGAAGTGTCAGGAGACGCTG	211	59	Clermont et al., 2000
	*yjaA*-R	ATGGAGAATGCGTTCCTCAAC			

### Multilocus sequence typing (MLST)

MLST was carried out on seven conserved housekeeping genes (*adk, fumC, gyrB, icd, mdh, purA*, and *recA*) according to the scheme of the *E. coli* MLST database (http://mlst.warwick.ac.uk/mlst/mlst/mlst/dbs/Ecoli/). The PCR products were purified with the QIAquick PCR purification kit (Qiagen, Germany), and double-strand sequenced using the ABI 3730 Automated DNA Analyzer (Applied Biosystems, USA). Each of the seven gene loci was assigned an allele number upon submission of the sequences to the *E. coli* MLST database. Allelic sequences with new variations gained new allele numbers. The allelic profile was used to generate a specific sequence type (ST) for each isolate.

### Phylogenetic analysis

For each strain, seven gene sequences were concatenated in the following order: *adk, fumC, gyrB, icd, mdh, purA*, and *recA*, to generate a 3423-bp DNA sequence for phylogenetic analysis. The concatamers were then aligned using the ClustalW program from the MEGA 6 software (http://www.megasoftware.net/). A Neighbor-Joining tree was constructed based on the maximum composite likelihood model with 1,000 bootstrap re-samplings using MEGA 6. To identify closely related genotypes, a minimum spanning tree (MST) based on different STs was constructed using Bionumerics software, version 4.6 (Applied Maths, Belgium).

### Statistical analysis

The presence of each virulence gene in aEPEC isolates from different sources were compared with each other by a two-tailed chi-square test or a Fisher's exact test using Epi Info software, version 3.5.3 (Maldonado et al., 2014). *P*-values < 0.05 were considered statistically significant.

## Results

### Serogroups and serotypes

The 228 aEPEC isolates belonged to 66 O serogroups with the exception of 17 (7.5%) strains being O-untypable (ONT) (Table 2, Table S1). Serogroup O51 was the most prevalent and was identified in 20 (8.8%) isolates, followed by O119 and O26, which were identified in 12 (5.3%) and 10 (4.4%) isolates, respectively. The remaining serogroups consisted of between 1 and 9 aEPEC strains. In diarrheal patients, animals and raw meats, the most common serogroups detected were O51, O119, and O76, respectively. No predominant serogroup was observed in strains obtained from healthy carriers.

**Table 2:** **Serotypes of 228 aEPEC isolates from different sources**.

**O:H serotype**	**Diarrheal patient**	**Healthy carrier**	**Animal**	**Raw meat**	**Total (%)**
O2:H40/H48/H49	3	0	0	3	6 (2.6)
O5:H19	0	0	1	0	1 (0.4)
O7:H11	1	0	0	0	1 (0.4)
O9:H19	1	0	0	0	1 (0.4)
O10:H2/HNT	1	0	0	2	3 (1.3)
O13(O129/O135):H11	2	2	0	0	4 (1.8)
O19:H9	1	0	0	0	1 (0.4)
O21:H6/H21	2	0	0	0	2 (0.9)
O23:H18	0	0	1	0	1 (0.4)
O26:H8/H11/HNT	2	1	6	1	10 (4.4)
O33:H6/H34	3	0	0	0	3 (1.3)
O34:H4/H9/HNT	1	1	1	0	3 (1.7)
O35:H19/H48	2	0	0	0	2 (0.9)
O37:H10	0	0	0	2	2 (0.9)
O40:H2/H19	2	0	0	0	2 (2.1)
O45:H2/H11/HNT	1	0	1	1	3 (1.3)
O49:H10	0	0	0	2	2 (0.9)
O50:H2	1	0	0	0	1 (0.4)
O51:H7/H21/H40/H49/HNT	16	0	3	1	20 (8.8)
O55:H7	1	0	0	0	1 (0.4)
O61:H2/H6/H10/H19	3	0	0	2	5 (2.2)
O63:H6	1	0	1	0	2 (0.9)
O70:H2/H11	1	0	0	1	2 (0.9)
O71:H11	0	0	0	1	1 (0.4)
O76:H6/H7	1	0	1	7	9 (3.9)
O82:H11	1	0	0	0	1 (0.4)
O85:H31	2	1	1	0	4 (1.8)
O86:H45	0	1	0	0	1 (0.4)
O88:H5/H8/H25	7	1	0	0	8 (3.5)
O91:H19	2	0	0	0	2 (0.9)
O92:H6	1	0	0	0	1 (0.4)
O101:H33/H4	4	0	0	0	4 (1.8)
O103:H4/H5/H8 /H21/H33	1	0	4	0	5 (2.2)
O107:H31	0	1	0	0	1 (0.4)
O107(O117):H40	1	1	0	0	2 (0.9)
O108:H9/H45	1	0	4	0	5 (2.2)
O109:H4/H19/H21/H34	6	0	0	0	6 (2.6)
O111:H9	1	2	0	0	3 (1.3)
O118:H5	1	0	0	0	1 (0.4)
O119:H4/H8/H21/H25	3	0	8	1	12 (5.3)
O120:H2/H10/H21/H45	1	0	3	0	4 (1.8)
O121:H33	0	0	1	0	1 (0.4)
O123:H11/H40/H45	2	0	1	1	4 (1.8)
O126:H19	3	0	0	0	3 (1.3)
O128:H2/HNT	5	0	1	0	6 (2.9)
O129:H11	1	1	0	0	2 (0.9)
O133:H10	2	0	0	0	2 (0.9)
O136:H21/H40	2	1	0	0	3 (1.3)
O137:H6	0	0	1	0	1 (0.4)
O138:H2/H48	3	2	0	0	5 (2.2)
O139:H14/H19	0	3	0	0	3 (1.3)
O141:HNT	0	0	1	0	1 (0.4)
O142:H34	1	0	0	0	1 (0.4)
O145:H2/H10/H28/H31/H34/H45	1	1	4	0	6 (2.6)
O152:H38	1	1	0	0	2 (0.9)
O156:H8/H21	1	0	0	1	2 (0.9)
O157:H5/H7/H33/H39	3	0	1	0	4 (1.8)
O158:H39	1	0	0	0	1 (0.4)
O164:H21	1	0	0	0	1 (0.4)
O167:H31	0	1	0	0	1 (0.4)
O170:H5/H8/H49	3	0	0	0	3 (1.3)
O171:H19	0	1	0	0	1 (0.4)
O172:H6	1	0	0	0	1 (0.4)
O177:H9/H11/H45	2	2	0	1	5 (2.2)
O180:H2	0	1	0	1	2 (0.9)
O182:H25	0	0	0	2	2 (0.9)
ONT:H2/H5/H6/H10/H16/H21/H25/H45/H48/H49/HNT	10	2	2	3	17 (7.5)
Total	121	27	47	33	228

Twenty-five different H-types were detected among the aEPEC strains (Table 2, Table S1). The most common were the flagellar types H11 (22 strains, 9.6%), H21 (20 strains, 8.8%), and H7 (20 strains, 8.8%). The H-type was not identified in 11 strains (4.8%). The most frequently detected H-types in diarrheal patients, animals and raw meats were H19, H21, and H7, respectively. No prominent H-type was observed in strains from healthy carriers.

### Virulence gene profiles

With the exception of *saa, subAB, espP, aat, aap*, and *pic*, all of the genes investigated were detected (Table 3, Table S1). The most frequently detected virulence gene among the 228 aEPEC isolates was *ehaC* (*n* = 183, 80.3%). The isolates from diarrheal patients, healthy carriers, animals, and raw meats exhibited a high prevalence of *ehaC*, with a detection rate of 82.6, 59.3, 83.0, and 84.8%, respectively. Significant difference in the overall presence of *ehaC* among the four different sources (*P* = 0.0350) was observed. *ehaC* was identified more often in isolates from diarrheal patients (82.6 vs. 59.3%, *P* = 0.0066), animals (83.0 vs. 59.3%, *P* = 0.0158) and raw meats (84.8 vs. 59.3%, *P* = 0.0159) than in isolates from healthy carriers.

**Table 3:** **Virulence genes in 228 aEPEC isolates recovered from different sources**.

**Genes**	**No. of aEPEC isolates carrying virulence gene (%)**				
	**Diarrheal patients**	**Healthy carriers**	**Animals**	**Raw meats**	**Total**
*efa1(lifA)*	36 (29.8)	0 (0)	14 (29.8)	9 (27.3)	59 (25.9)
*nleB*	50 (41.3)	2 (7.4)	22 (46.8)	22 (66.7)	96 (42.1)
*nleE*	53 (43.8)	2 (7.4)	24 (51.1)	20 (60.1)	99 (43.4)
*set/ent*	50 (41.3)	2 (7.4)	21 (44.7)	22 (66.7)	95 (41.7)
*lpfA*	66 (54.5)	11 (40.7)	31 (66.0)	16 (48.5)	124 (54.4)
*toxB*	12 (9.9)	1 (3.7)	6 (12.8)	1 (3.0)	20 (8.8)
*iha*	19 (15.7)	5 (18.5)	7 (14.9)	5 (15.2)	36 (15.8)
*paa*	44 (36.4)	4 (14.8)	20 (42.6)	21 (63.6)	89 (39.0)
*ehxA*	3 (2.5)	2 (7.4)	7 (14.9)	9 (27.3)	21 (9.2)
*astA*	13 (10.7)	5 (18.5)	9 (19.1)	3 (9.1)	30 (13.2)
*pet*	13 (10.7)	8 (29.6)	0 (0)	0 (0)	21 (9.2)
*cdt*	1 (0.8)	3 (11.1)	6 (12.8)	2 (6.1)	12 (5.3)
*katP*	9 (7.4)	3 (11.1)	5 (10.6)	6 (18.2)	23 (10.1)
*ehaC*	100 (82.6)	16 (59.3)	39 (83.0)	28 (84.8)	183 (80.3)
*yja*	44 (36.4)	17 (63.0)	18 (38.3)	14 (42.4)	93 (40.8)

Among the adhesion genes investigated, *lpfA* exhibited the highest prevalence in animal strains (66.0%), followed by diarrheal patient strains (54.5%), raw meat strains (48.5%), and healthy carrier strains (40.7%). However, we could not detect a significant difference in the overall presence of *lpfA*. The *paa* gene was identified with the highest prevalence in raw meat strains (63.6%), followed by animal strains (42.6%), diarrheal patient strains (36.4%), and healthy carrier strains (14.8%). There was a statistically significant difference in the overall presence of *paa* between the four different sources (*P* = 0.0013). *paa* was identified more often in isolates from raw meats (63.6 vs. 14.8%, *P* < 0.0001), animals (42.6 vs. 14.8%, *P* < 0.0122) and diarrheal patients (36.4 vs. 14.8%, *P* < 0.0225) than in strains from healthy carriers. Compared to *lpfA* or *paa*, the prevalence of *iha* and *toxB* was lower in aEPEC strains (15.8 and 8.8%, respectively).

We also investigated four genes located on OI-122 (*efa1* (*lifA*), *set/ent, nleB*, and *nleE*), and the phylogenetic marker *yjaA* gene. Of the 121 strains from diarrheal patients, 36 were *efa1* (*lifA*)-positive (29.8%), 50 were *nleB*-positive (41.3%), 53 were *nleE*-positive (43.8%), 50 were *set/ent*-positive (41.3%), and 44 were *yjaA*-positive (36.4%). Of the 27 strains from healthy individuals, none were *efa1* (*lifA*)-positive, two strains were *nleB-, nleE-*, and *set/ent*-positive (7.4%), respectively, and 17 were *yjaA*-positive (63.0%). Among the 47 strains from animals, 14 were *efa1* (*lifA*)-positive (29.8%), 22 were *nleB*-positive (46.8%), 24 were *nleE*-positive (51.1%), 21 were *set/ent*-positive (44.7%), and 18 were *yjaA*-positive (38.3%). Of the 33 strains from raw meats, 9 were *efa1* (*lifA*)-positive (27.3%), 22 were *nleB*-positive (66.7%), 20 were *nleE*-positive (60.1%), 22 were *set/ent*-positive (66.7%), and 14 were *yjaA*-positive (42.4%). The complete OI-122 was detected in 35 (28.9%), 0, 13 (27.7%), and 6 (18.2%) strains from diarrheal patients, healthy carriers, animals and raw meats, respectively (Table S1). In contrast, incomplete OI-122 was observed in 19 (15.7%), 2 (7.4%), 12 (25.5%), and 18 (54.5%) strains from diarrheal patients, healthy carriers, animals and raw meats, respectively. aEPEC strains carrying OI-O122 genes with different combinations but lacking *efa1 (lifA)* were also found. There were statistically significant differences in the overall presence of *efa1* (*lifA*), *nleB, nleE*, and *set/ent* genes among the four different sources (*P* = 0.0130, *P* = 0.0001, *P* = 0.0002, and *P* = 0.0001, respectively). Strains from diarrheal patients had significantly more *efa1* (*lifA*) (29.8% vs. 0, *P* = 0.0002), *nleB* (41.3 vs. 7.4%, *P* = 0.0004), *nleE* (43.8 vs. 7.4%, *P* = 0.0002) and *set/ent* (41.3 vs. 7.4%, *P* = 0.0004) genes than strains obtained from healthy carriers. Meanwhile, *efa1* (*lifA*), *nleB, nleE*, and *set/ent* genes were each found to be more prevalent in aEPEC strains isolated from animals or raw meats than strains from healthy carriers. *yjaA* was more frequently observed among healthy carrier strains than in diarrheal patient isolates (63.0 vs. 36.4%, *P* = 0.0105) and in animal isolates (63.0 vs. 38.3%, *P* = 0.0354).

Among the virulence genes linked to the EHEC pO157 plasmid, *ehxA* and *katP* were detected at high prevalence in raw meat strains (27.3 and 18.2%, respectively), while these genes were detected at low prevalence in diarrheal patient strains (2.5 and 7.4%, respectively). There was a statistically significant difference in the overall presence of *ehxA* among the four different sources (*P* = 0.0001). It was detected more often in isolates from raw meats than in strains from diarrheal patients (27.3 vs. 2.5%, *P* = 0.0000) and healthy carriers (27.3 vs. 7.4%, *P* = 0.0474). However, no significant difference was observed between raw meat strains and animal strains. In contrast, *cdt* was present at higher prevalence in animal strains (12.8%) and healthy carrier strains (11.1%) than in raw meat strains (6.1%) and diarrheal patient strains (0.8%). Furthermore, *astA* and *pet* were observed at higher prevalences in healthy carrier strains (18.5 and 29.6%, respectively) than in diarrheal patient strains (both were 10.7%).

### Multilocus sequence typing

**Figure 1:**
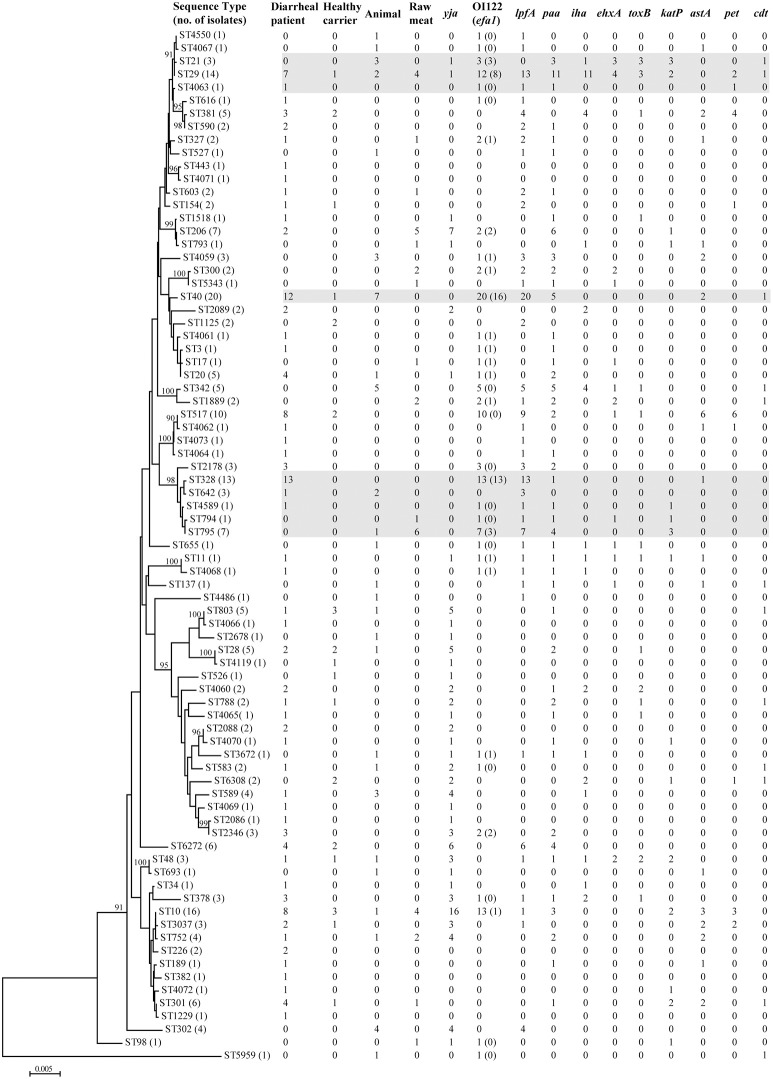
**Phylogenetic relationships and virulence profiles of the 79 STs among the 228 aEPEC isolates**. An unrooted phylogenetic tree was constructed by the neighbor-joining algorithm based on the Maximum Composite Likelihood model of nucleotide substitution. Bootstrap values greater than 90% based on 1000 replications are given at the internal nodes. STs highlighted in gray were the most prominent STs containing aEPEC isolates and harbored genes located on OI-122 (*nleB, nleE, set/ent*, or *efa1* (*lifA*)) and *lpfA*.

Seventy-nine different sequence types (STs) were detected among 228 aEPEC isolates (Figure 1, Table S1). In diarrheal patients, healthy carriers, animals and raw meats, 53, 17, 26, and 15 STs were detected, respectively. The most frequently detected ST was ST40 (20/228, 8.8%), with 12 strains from diarrheal patients, seven from animals and one from a healthy carrier. The second predominant ST, ST10, was identified from eight diarrheal patient isolates, four raw meat isolates, three healthy carrier isolates, and one animal isolate. ST29 and ST328 comprised 14 and 13 isolates, respectively. Another 35 STs comprised 2-10 isolates, while 40 STs had only one single isolate. Among 25 STs that included three or more isolates, four STs (ST328, ST378, ST2178, and ST2346) were detected only in diarrheal patient isolates, and four STs (ST342, ST302, ST21, and ST4059) were present only in animal isolates.

**Figure 2:**
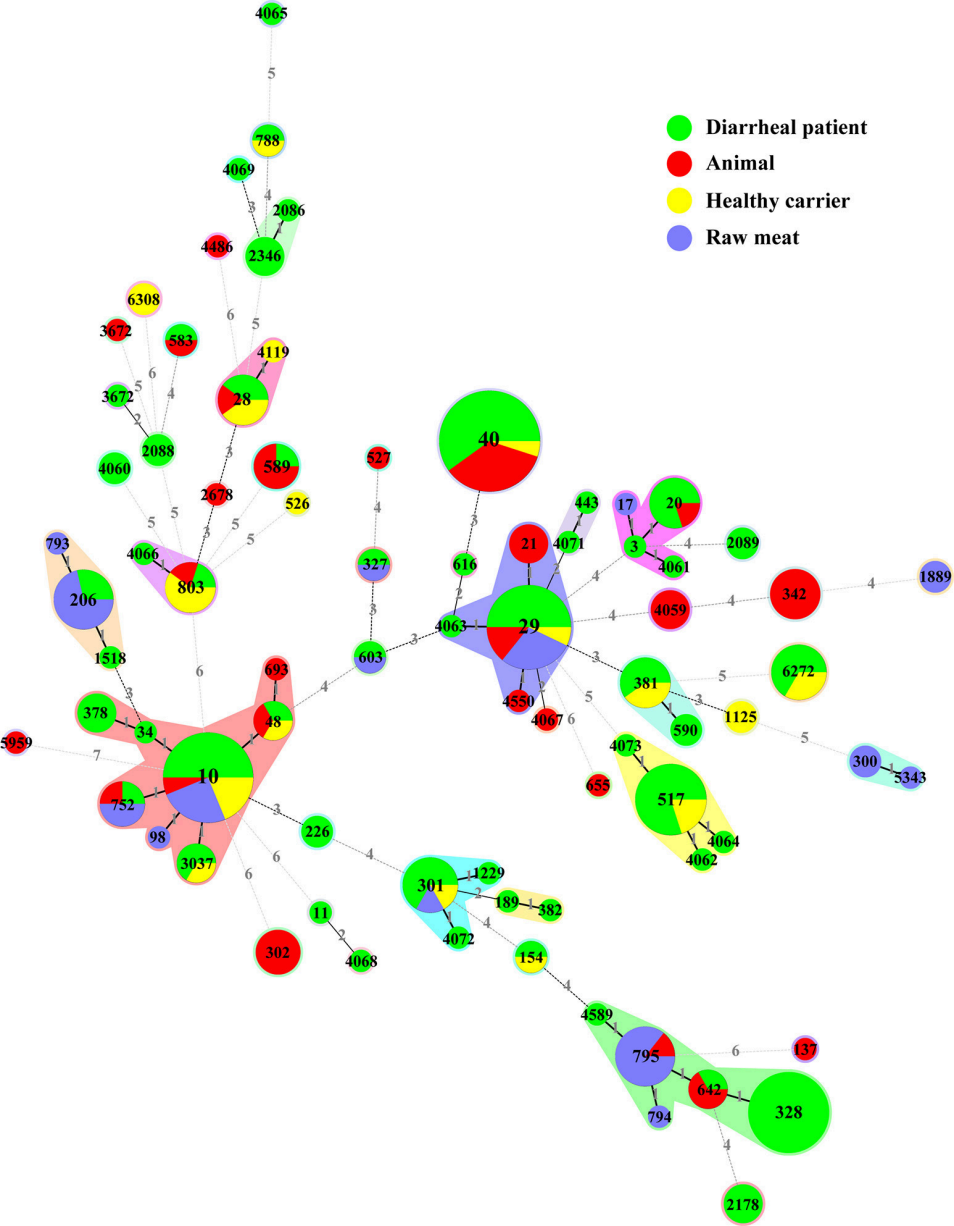
**Minimal spanning tree of the 228 aEPEC isolates based on STs**. Each circle represents a given ST with size proportional to the number of isolates. The colors for the slices of the pie represent different sources of isolates: diarrheal patient in green, animal in red, healthy carrier in yellow and raw meat in purple. The numbers on connecting lines show the number of allelic difference between two STs. The number in the circle is the ST number.

We constructed an MST containing 79 STs obtained in our study (Figure 2, Table S1). They were clustered into 34 singletons and 14 non-overlapping groups or clonal complexes (CC). Most STs differed from each other by 2 or more alleles. Only two STs (i.e., ST10 and ST29) contained isolates from all sources (diarrheal patients, health carriers, animals and raw meats).

### Prevalence of virulence genes among different STs

There were quite differences in the number of virulence genes among different isolates, ranging from 0 to 12 genes per isolate. Two isolates from diarrheal patients belonging to ST11 (O157:H7) and ST29 (O7:H11) contained 12 and 11 virulence genes, respectively (Figure 1, Table S1).

Genes located on the pathogenicity island OI-122 (*nleB, nleE, set/ent*, or *efa1* (*lifA*)) were identified in isolates belonging to 33 different STs. Complete OI-122 was detected in 54 isolates belonging to 16 different STs. The most prominent STs were ST40 (15 isolates), ST328 (13 isolates), and ST29 (8 isolates). The most common clonal complexes were CC278 (16 strains), CC40 (15 strains), and CC29 (11 isolates). The presence of the OI-122 genes was associated with different combinations of other virulence genes. Among the 54 isolates harboring the complete OI-122, 51 strains were *ehaC* positive with 42 strains being *lpfA* positive, while *paa, ehxA, iha, toxB, katP, astA*, and *cdt* were present in 26, 9, 9, 6, 6, 4, and 1 isolates, respectively (Figure 1, Table S1).

Three variants of the *lpfA* gene (*lpfA*_*O113*_, *lpfA*_*O157/OI–154*_, and *lpfA*_*O157/OI–141*_) were detected in 40 different STs, which contained 124 isolates. The most prominent STs were ST40 (all carrying *lpfA*), ST328 (all carrying *lpfA*) and ST29 (all except one carrying the *lpfA* gene). In contrast, for the 32 isolates contained in CC10, the *lpfA* gene was only detected in four isolates without any of the OI-122 genes.

## Discussion

The present study is the first detailed molecular profiling study of aEPEC strains from China. Serotyping results showed that 66 serogroups were identified, most of which were non-classical serogroups (Hernandes et al., 2009). The WHO has recognized 12 O serogroups as the classical EPEC serogroups, i.e., O26, O55, O86, O111, O114, O119, O125, O126, O127, O128, O142, and O158. Nine classical serotypes were found in our study, which contained 38 (16.7%) aEPEC isolates. They were not collectively more prevalent than the remaining serogroups. In fact, the most frequently detected serogroup of aEPEC reported so far is O51, followed by O145, O26, O55 and O111, and O119 (Hernandes et al., 2009). Globally, there are various studies showing that aEPEC has different serogrous, such as O76 (Møller-Stray et al., 2012), O127 (Hao et al., 2012), O119, O26, and O88 (Jenkins et al., 2003). In this study, 7.4% of aEPEC isolates were O untypable, which is much lower than that reported by others (Blanco et al., 2006; Monaghan et al., 2013; Zhang et al., 2016). Blanco et al. found that 110 EPEC strains were typed to 43 O serogroups and 69 serotypes with 44 new serotypes and only 13% could be assigned to classical EPEC serotypes (Blanco et al., 2006). So, the vast diversity of serotypes suggested that serotyping alone is insufficient for the detection of aEPEC. To better characterize aEPEC strains, it is necessary to combine the phenotypic and genotypic information.

Virulence gene profiling is essential in determining the pathogenic potential of these strains. In our investigation, statistically meaningful differences were observed among the distributions of OI-122 genes *efa1* (*lifA*), *nleB, nleE, set/ent* and the phylogenetic marker gene *yjaA*. Afset et al. observed that *efa1* (*lifA*) was the gene with the strongest statistical association with diarrhea (Afset et al., 2006). The other three genes were also found to be linked to diarrhea, while gene *yjaA* was negatively associated with diarrhea. Our results showed that OI-122 genes *efa1* (*lifA*), *nleB, nleE, set/ent*, and the phylogenetic marker gene *yjaA* were associated with diarrhea among the aEPEC isolates studied, which is consistent with the results detected by Afset and colleagues (Afset et al., 2006). Furthermore, *efa1* (*lifA*), *nleB, nleE*, and *set/ent* were found to be more prevalent in aEPEC strains isolated from animals or raw meats than in strains from healthy carriers. These findings indicated that aEPEC strains from different sources might carry different virulence gene profiles. The presence of aEPEC strains carrying OI-122 genes in animals and raw meats might pose a zoonotic risk to humans. This finding also supports the concept that animals are the sources of aEPEC infection in humans (Wang et al., 2013).

The *paa* gene is frequently detected in porcine EPEC. Paa is involved in intimate attachment of the bacteria to enterocytes and causes post-weaning diarrhea in pigs (Batisson et al., 2003). In accordance with previous studies, the positive association between *paa* and diarrhea was also observed in our study. Balière et al. (2016) observed a significantly higher (60%) occurrence of *paa* in EPEC isolates from environment samples, which is similar to the frequency we observed in raw meat isolates (63.6%) in this study. *paa* was much more frequently detected in strains isolated from raw meats than from other sources, and was especially higher than strains from healthy carriers (14.8%). Similarly, the gene *ehxA* that encodes the EHEC hemolysin, was also found at a higher frequency among aEPEC from raw meats, although we could not confirm the positive association of *ehxA* with diarrhea (Afset et al., 2006; Scaletsky et al., 2009) in this study. In a previous study, an outbreak caused by aEPEC following the consumption of contaminated food or water was reported (Wedley et al., 2013). Thus, the high presence of aEPEC possessing *paa* or *ehxA* in raw meats indicates that these isolates could cause disease if ingested by humans, and therefore, remains a serious public health threat to consumers.

In a previous study, the virulence marker *astA* was significantly associated with diarrhea (Dulguer et al., 2003). In Japan, a waterborne outbreak of diarrhea was found to be associated with aEPEC strains carrying the *astA* gene (Yatsuyanagi et al., 2003). However, a negative statistical association with diarrhea has also been reported (Afset et al., 2006). In the current investigation assessing 228 isolates, no correlation was confirmed, in agreement with another study (Scaletsky et al., 2009).

Autotransporter (AT) proteins are often associated with virulence attributes such as adherence, biofilm formation, and toxicity and these proteins have been identified in both tEPEC and aEPEC (Abreu et al., 2013). Several studies have investigated the presence of AT genes in EPEC (Abreu et al., 2013; Balière et al., 2016). In the present study, four AT genes (*ehaC, espP, pet*, and *pic*) were detected. Among these genes, the most frequent gene observed in our isolates was *ehaC*, which was detected at frequencies similar to those observed by Abreu and colleagues (Abreu et al., 2013). However, it was found more often in isolates from diarrheal patients, animals and raw meats than in isolates from healthy carriers. Our hypothesis is that gene *ehaC* might play a role in aEPEC infection.

Afset et al. assessed the phylogenetic group and association with diarrhea of 56 aEPEC strains and found that phylogenetic groups B1 and D were weakly associated with diarrhea (Afset et al., 2008). Wang et al. observed that phylogroup B1 was detected more frequently among patient strains than healthy carrier strains (Wang et al., 2013). Furthermore, they also demonstrated that the virulence group Ia comprising the *efa1* (*lifA*) gene and/or *lpfA* genes but without the *yjaA* gene, was also detected more frequently in clinical isolates compared with healthy carriers. A gene cluster encoding enzymes involved in O-antigen synthesis was found to be significantly associated with lethal infections (Donnenberg et al., 2015). The pathogenesis of aEPEC also seems to be related to the serotypes (Hu and Torres, 2015). In our investigation, a different method of MLST was employed for phylogenetic group determination. There was a trend for the retention of complete OI-122 and the relationship with specific STs or clonal complexes and serotypes. Genes of OI-122 or *lpfA* were more frequently detected in ST40 strains with O119:H21 or O109, ST328 strains with O51:H7 or O88:H25, ST795 strains with O76:H7, and ST29 or ST21 strains with O26. These aEPEC strains that were isolated from diarrheal patients, animals and raw meats harbored genes reported to be associated with human diarrhea and might be considered more potentially pathogenic. It has been reported that many virulence genes are present on mobile elements, such as, plasmids, PAIs, or phages and are highly interchangeable among different bacterial strains through horizontal transfer (Hacker and Kaper, 2000). The results that some virulence genes are present more often in specific STs and serotypes than other members suggest that some STs and serotypes of aEPEC might have different genomic backgrounds which permit the acquisition and expression of virulence factors. However, the reason for this is unknown. The detection of such aEPEC strains by subgroup demands further attention. Molecular assessment combining phylogenetic analysis and virulence gene profiling might be employed to establish the pathogenic potential of aEPEC strains.

However, in the present study, there were fewer isolates from raw meats and healthy carriers than from diarrheal patients. Unequal sample sizes and bias in data collection might exist. Nevertheless, this was the most comprehensive data analyzing aEPEC isolates in China. Also, the results did show the vast diversity of the aEPEC isolates from different sources in China.

In conclusion, aEPEC strains in China are heterogeneous in serotypes, virulence gene profiles and phylogenies. It is possible that different aEPEC strains might have different pathogenic potential. The aEPEC strains which were more potentially pathogenic seemed to be related to specific STs or clonal complexes and serotypes.

## Author contributions

YXu and YX designed the project; YJ, BH, HW, RF, and SF carried out the sampling work; YXu, XB, SF, and RF carried out the experiments and generated data; YXu and YX analyzed data and drafted the manuscript. All authors have read and approved the final version of the manuscript.

### Conflict of interest statement

The authors declare that the research was conducted in the absence of any commercial or financial relationships that could be construed as a potential conflict of interest.
